# Analyzing Personal Happiness from Global Survey and Weather Data: A Geospatial Approach

**DOI:** 10.1371/journal.pone.0153638

**Published:** 2016-04-14

**Authors:** Yi-Fan Peng, Jia-Hong Tang, Yang-chih Fu, I-chun Fan, Maw-Kae Hor, Ta-Chien Chan

**Affiliations:** 1 Department of Computer Science, National Chengchi University, Taipei, Taiwan, Republic of China; 2 Research Center for Humanities and Social Sciences, Academia Sinica, Taipei, Taiwan, Republic of China; 3 Institute of Sociology, Academia Sinica, Taipei, Taiwan, Republic of China; 4 Institute of History and Philology, Academia Sinica, Taipei, Taiwan, Republic of China; 5 School of Informatics, Kainan University, Taoyuan, Taiwan, Republic of China; University of Rochester, UNITED STATES

## Abstract

Past studies have shown that personal subjective happiness is associated with various macro- and micro-level background factors, including environmental conditions, such as weather and the economic situation, and personal health behaviors, such as smoking and exercise. We contribute to this literature of happiness studies by using a geospatial approach to examine both macro and micro links to personal happiness. Our geospatial approach incorporates two major global datasets: representative national survey data from the International Social Survey Program (ISSP) and corresponding world weather data from the National Oceanic and Atmospheric Administration (NOAA). After processing and filtering 55,081 records of ISSP 2011 survey data from 32 countries, we extracted 5,420 records from China and 25,441 records from 28 other countries. Sensitivity analyses of different intervals for average weather variables showed that macro-level conditions, including temperature, wind speed, elevation, and GDP, are positively correlated with happiness. To distinguish the effects of weather conditions on happiness in different seasons, we also adopted climate zone and seasonal variables. The micro-level analysis indicated that better health status and eating more vegetables or fruits are highly associated with happiness. Never engaging in physical activity appears to make people less happy. The findings suggest that weather conditions, economic situations, and personal health behaviors are all correlated with levels of happiness.

## Introduction

Happiness is one of the most important human emotions and a key predictor of many important life outcomes. Several studies have examined how factors in the external environment and human behaviors affect happiness [1, 2]. Those factors can be roughly divided into either macro or micro effects. The former include weather effects, such as temperature [3], sunlight [4, 5], seasonal climate change [6], and the societal socio-economic environment [7], while the latter include individuals’ demographic factors such as gender [8], age [4], and personal health behaviors like smoking [9], exercise [10], and eating more fruits and vegetables [11]. Studies have indicated that several factors simultaneously affect people’s happiness, and the variations occur in different persons, cities, and countries. One previous study pointed out that climate change might alter the distribution of happiness among countries [1]. The spatiotemporal resolution of the weather data, however, might have deeply affected this inference.

To examine how such a spatiotemporal perspective may help advance our knowledge about happiness, we integrate global survey data with various corresponding weather conditions. In particular, we expand the spatial resolution from the country level to the city level, and the temporal resolution from the monthly level to the daily level. Short-term weather effects also might be correlated with happiness. While the preexisting world database of happiness archives several research findings on the subjective enjoyment of life in the form of summarized statistics for each country [12], this study focuses on individual-level survey data across 32 countries. Such survey data facilitate multivariate analyses that control for personal factors and variations within countries. Because happiness is such a complex issue linked to various personal and societal circumstances, it would be even more revealing and challenging to further enrich such individual-level analyses in light of macro-level factors.

In this study, we aim to examine the macro and micro effects on happiness from a global perspective. To cover such different circumstantial factors, we integrated international social survey data from the International Social Survey Programme (ISSP): Health and Health Care—ISSP 2011 [12] and weather data called Global Summary of the Day (GSOD) from global meteorological stations [13], from the National Oceanic and Atmospheric Administration (NOAA). Using a geospatial method, we not only could connect these two highly different types of data, but also incorporate other factors that may be linked to happiness, such as the cities’ economic development and geo-information. By exploring how weather, economic situations, and personal health behaviors are associated with happiness, our study also points to the potential power and usefulness of combining two seemingly distant and divergent global datasets.

## Materials and Methods

### Ethics and Survey data

The ISSP survey data that we used are available to the public from the GESIS data archive (https://dbk.gesis.org/dbksearch/download.asp?db=E&id=56356). None of the databases that we used include identifiable personal information, thus informed consent was not necessary. Because the datasets we used in this study are all publicly available, furthermore, no approval from an institutional review board (IRB) was needed. The dataset was the 2011 ISSP module on health and health care, conducted from 2011 to 2013 by 32 countries. Because the survey data in three countries (Japan, Norway, and South Africa) lacked the specific dates of interviews, which made it impossible to link the two global data sets for these countries, we constructed the weather data to correspond to the survey data for the remaining 29 countries (see S1 Table for the list of member countries).

The purpose of the module was to evaluate healthcare systems, personal health, and health insurance in various national contexts. Among the survey topics, satisfaction with life was measured by respondents’ degree of self-reported happiness. Using it as our main focus, we adopt this measure of happiness as our dependent variable. In the original ISSP questionnaire, the degree of happiness was self-reported on a 7-point scale of "completely happy," "very happy," "fairly happy," "neither happy nor unhappy," "fairly unhappy," "very unhappy," and "completely unhappy." The only exception was China, where happiness was measured on a 5-point scale from "very happy," "quite happy," "I’m not sure, neither happy nor unhappy," and "quite unhappy" to "very unhappy." Due to differences in the response scales, we decided to first use data from 28 countries (all but China), then analyze the data from China separately. Using these two different datasets enabled us to better validate and compare the results.

### Meteorological data

To elucidate the relationship between weather and happiness, we collected daily weather data from meteorological stations around the world. There are 28,014 meteorological stations with spatial coordinates throughout the world that can be accessed at the website (ftp://ftp.ncdc.noaa.gov/pub/data/gsod) maintained by the National Oceanic and Atmospheric Administration (NOAA). The daily Global Summary of the Day (GSOD) includes the temperature, dew point, visibility, wind speed, and so on from each station. We extracted the meteorological data from stations located in the cities with ISSP’s samples. This data-extraction procedure allowed more precise matches between the survey samples and local weather conditions.

### Administrative area data

When analyzing ISSP data, we used the city as our basic spatial unit. To compute the geographic location of cities, we needed to identify the administrative boundaries around the world. We used a global digital map from the publicly available GADM database of Global Administrative Areas (http://www.gadm.org/) to locate such boundaries [14].

### Methods

Our study relied heavily on both data processing and statistical analysis. Detailed data processing allowed us to organize, filter, and compare both the survey data and weather data that we retrieved. This procedure could be further divided into “data preprocess,” “geocoding process,” “integration process,” and “data fetching.”

#### Data preprocess

To locate the cities where the survey respondents lived, and to identify the corresponding meteorological stations in or close to these cities, we needed to geo-process both the survey data and the meteorological data. The purpose of this step was to extract useful information for geocoding. First, the names of all 32 countries in the ISSP data set were easily identifiable. All respondents also reported the cities in which they lived at the time of the interview. By geocoding those two variables, we obtained every respondent’s spatial information.

The same process was applied to the meteorological stations as well. In this study, we selected four meteorological variables as our explanatory variables, including temperature, dew point, visibility, and wind speed for the corresponding stations for the 2011–2013 study period. In total, we used 1,517 meteorological stations in this study. Due to the different date of establishment in each station, some weather records were not available for specific survey dates. Although the NOAA provides the spatial coordinates of every station, no data linkage is readily available between the stations and GSOD. As a result, we merged these two data sources based on the unique station numbers.

#### Geocoding process

The ISSP data included 55,081 survey records covering 32 countries, which needed to be geocoded. We used two steps to reduce the complexity of the geocoding process. Based on city of residence, we first classified all records into 510 cities or regions. We then used the Google Maps Geo-coding API [15] to derive most city coordinates. To validate the geocoding accuracy, we checked the results manually. After correcting missing or erroneous geocoding results, we managed to obtain the spatial coordinates for all survey records.

**Fig 1:**
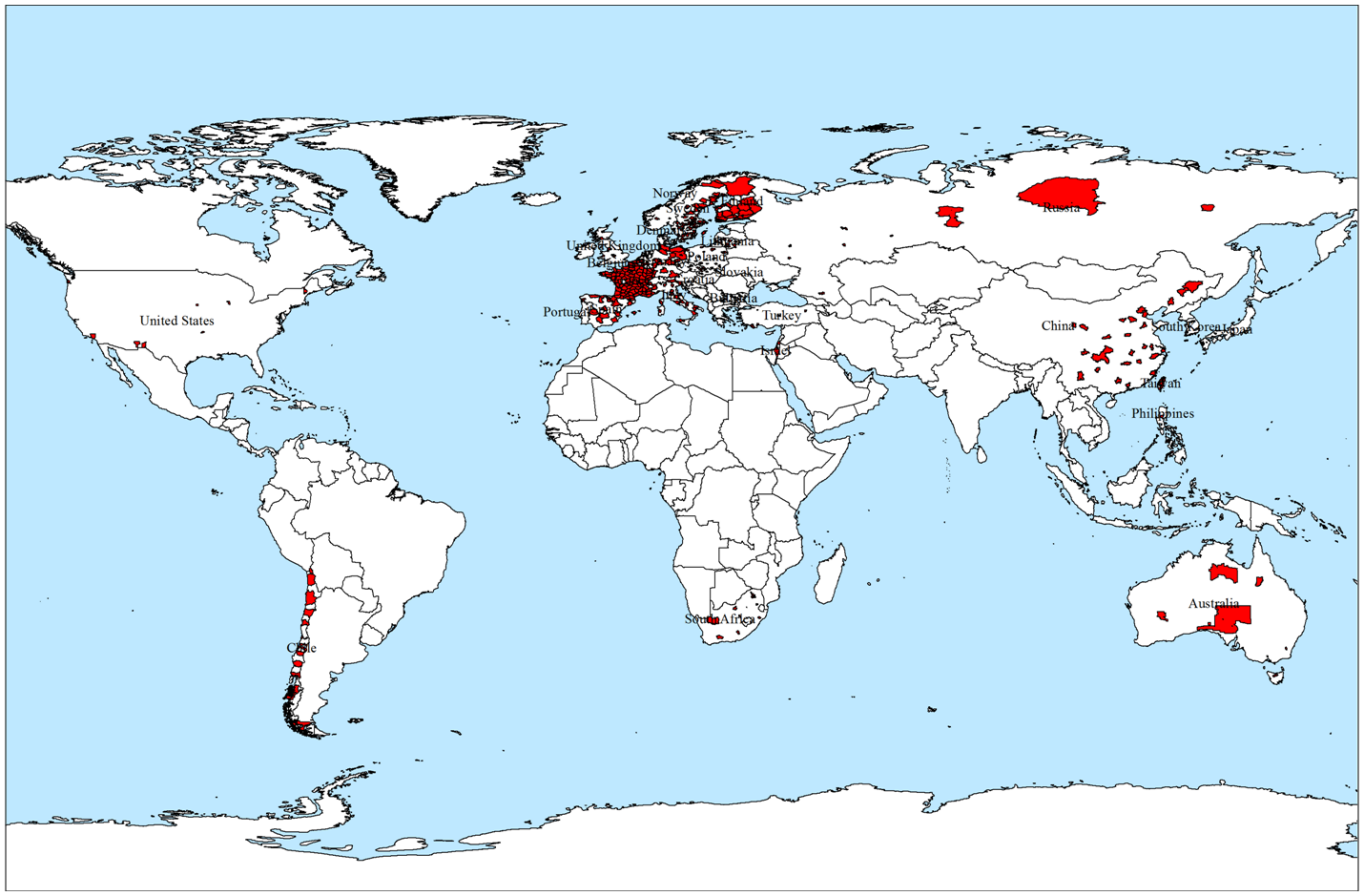
The spatial distribution of the cities derived from survey data.

The spatial information in the NOAA and the ISSP is stored in different formats. In the NOAA dataset, weather stations were marked by latitude and longitude, without exact city names. In contrast, the locations in the ISSP were presented by the cities’ names, some of which were written in languages other than English. To minimize these discrepancies, we used the international administrative area data from the GADM as our base map. We spatially integrated these two different types of source data into one common map using the spatial join function in ArcGIS (ArcMap, version10.2; ESRI Inc., Redlands, CA, USA). The integration helped produce a map with the respondents and meteorological stations connected to each city, allowing us to map the spatial distribution of happiness from the survey data at the city level throughout the world. Those areas marked in red shown in Fig 1 are the cities that came from survey data.

#### Integration process

We needed the exact date of the interview to integrate the survey data with the weather data that we extracted from the GSOD. Due to this requirement, we had to remove 4,866 records with incomplete survey dates in the ISSP survey data, including those from Norway and South Africa, which only recorded month and year. We also excluded Japan from our analysis, because all respondents in this country were coded with the same date.

#### Data fetching

To evaluate the short-term weather effects, we set three different periods of the date and computed the average effects of weather variables in each period. The three different periods of the date are 2 days, 4 days, and 8 days, which refer to the survey date and its past 1 day, 3 days, and 7 days, respectively. The three periods of average weather variables were then analyzed into three separate models.

The models also take other macro-level factors into account. GDP, for example, was used to represent the overall economic situation. The survey data in each country ended in different years. Therefore, it was not suitable to use GDP data in one fixed year. To solve this problem, we used the difference in GDP between the survey year and the previous year. In other words, we focused on the increase or decrease of the GDP instead of the absolute value of the GDP itself.

**Fig 2:**
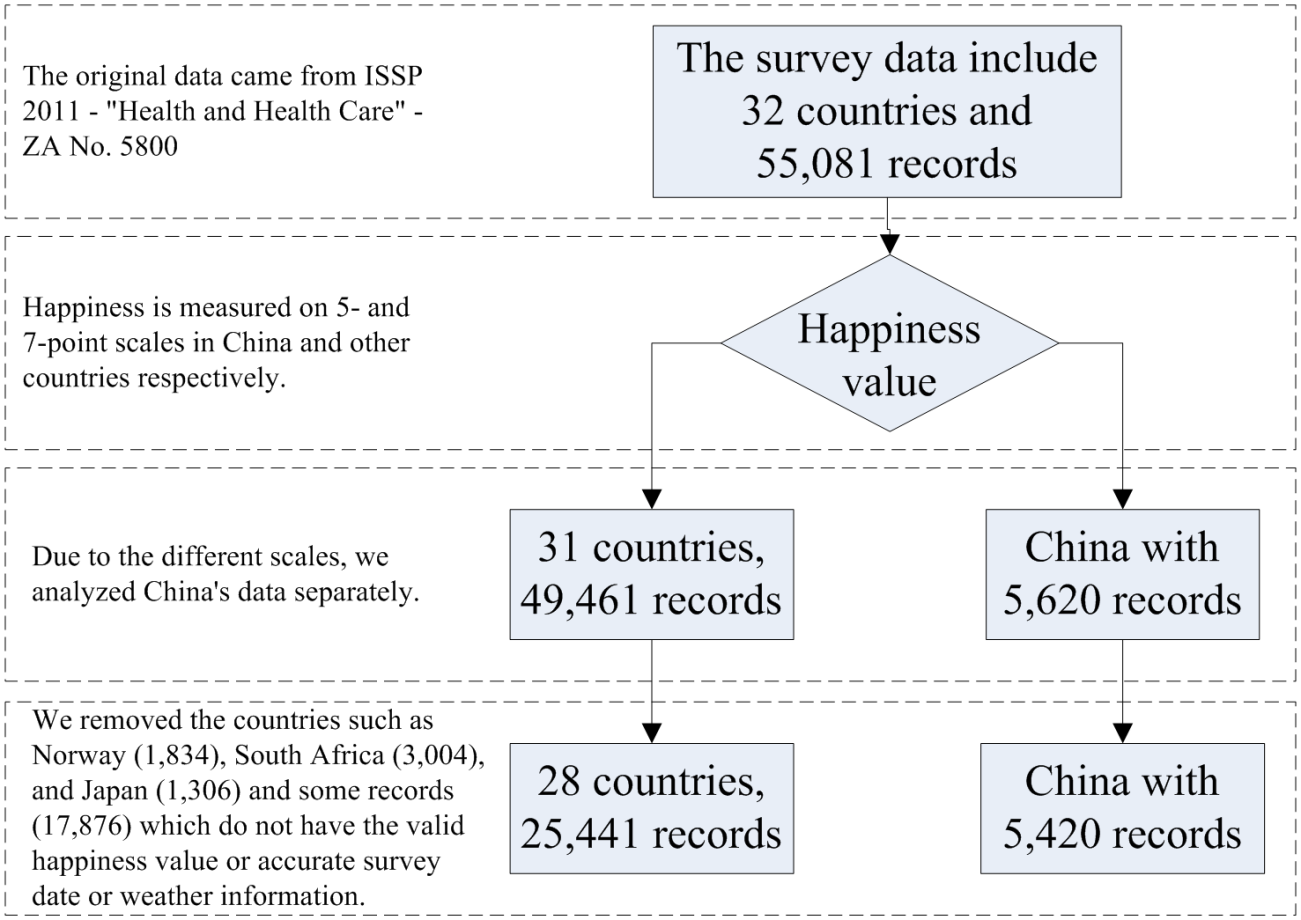
The workflow of data processing.

Fig 2 shows the workflow of data processing and the number of records in every step. After the first part of data processing, we obtained the filtered ISSP data and the corresponding weather data and GDP data.

### Statistical analysis

This study used quantitative methods that allowed a reliable inference of associations between weather conditions and personal happiness. The software used in processing the statistical data is the IBM SPSS Statistics version 20. The programming syntax used in SPSS is provided in S2 Table. The research question is whether and which weather conditions are significantly correlated with personal happiness across countries and regions of the world. We treated happiness as ordinal, under the assumption that degrees of happiness have a natural order (low to high), with the quantitative difference between adjacent categories not exactly known. We chose ordinal logistic regression analysis, because it could properly measure the dependent variable on an ordinal scale. An ordinal logistic regression model can be seen as an extension of logistic regression. While the latter evaluates binary dependent variables, ordinal logistic regression models take into account dependent variables with more than two response categories ordered in a logical sequence, e.g., from very unhappy to very happy.

In each of the models, we used the following parameters as the key weather variables: mean temperature (°F), mean dew point (°F), mean visibility (miles), mean wind speed (knots), and the elevation of the city (hectometers). To calculate each city’s elevation, we first located the center coordinate of the city from the GADM by ArcGIS, followed by Google Maps Elevation API [16].

For individual-level variables from ISSP survey data, we included gender, age, self-reported health status, the frequency of visits to a doctor in the past 12 months, smoking history, and the frequencies of drinking alcohol, physical activity, and eating fresh fruits or vegetables.

After fitting a statistical model, it is important to determine whether all the necessary model assumptions are valid before performing inferencial procedures. If there are any violations, subsequent inferential procedures may be invalid, and if so, the conclusions would be faulty. Therefore, it is crucial to perform appropriate model diagnostics. All relevant model diagnostic procedures for the ordinal logistic regressions used in this study are provided in greater detail in the S1 File.

## Results

### Descriptive statistics

**Fig 3:**
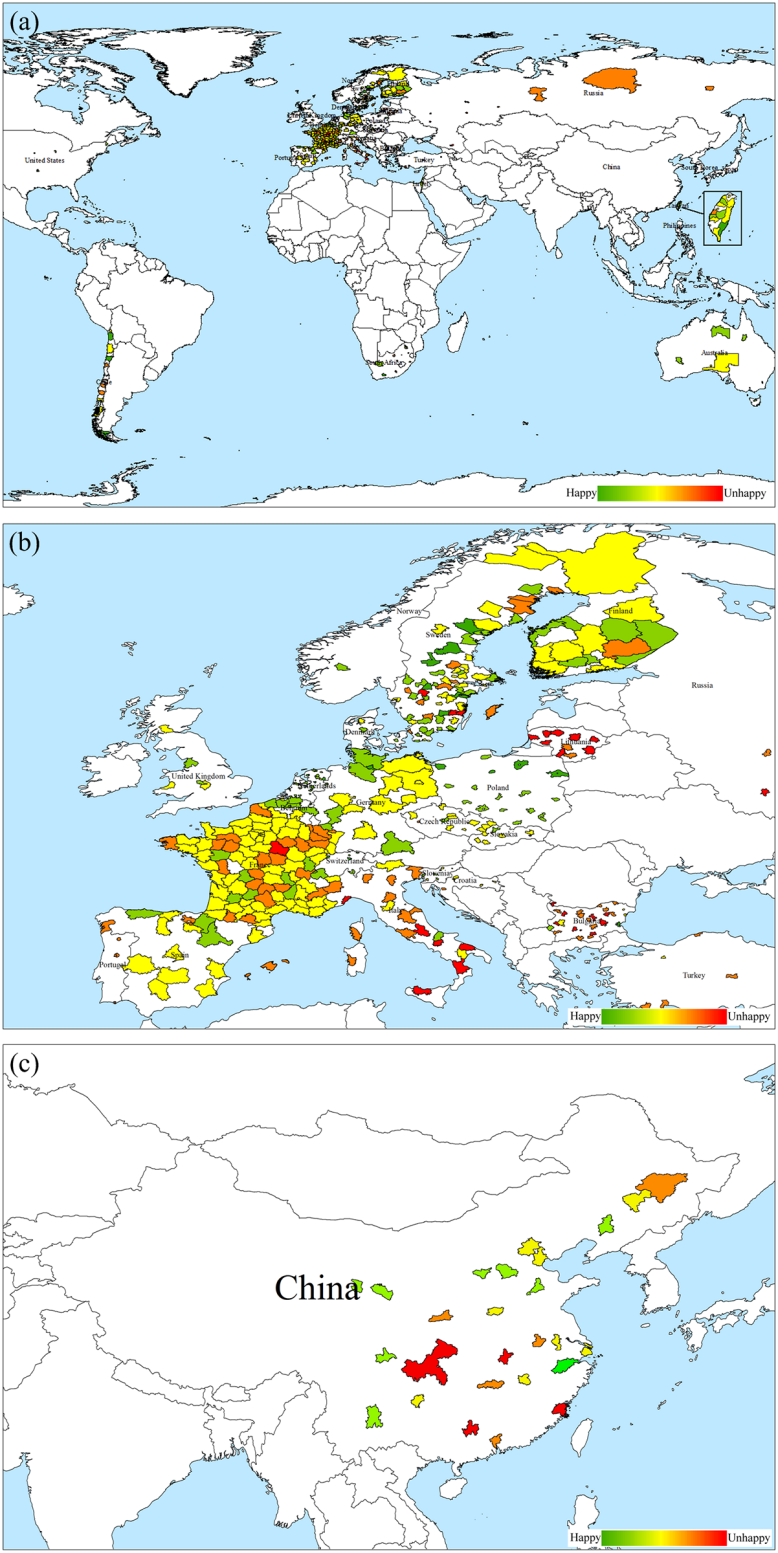
The happiness map. (a) The happiness map of the world except China. (b) The happiness map of Europe (c) The happiness map of China.

Fig 3 shows the distribution of happiness in different survey areas. Green indicates happier and red means unhappier. Overall, residents in Europe were happier than those in other areas, as shown in Fig 3a. Those living in northern Europe were happier than those in southern Europe (Fig 3b). In China, however, there is not an obvious spatial pattern about happiness (Fig 3c). The descriptive statistics of the macro and micro variables among the 28 countries and China are listed in Tables 1 and 2, respectively. Among the macro variables, temperature ranged from -17.41°F to 87.6°F in the 28 countries, and from 26.62°F to 90.32°F in China. The range of visibility was from 0 mile to 31.76 miles in the 28 countries, and from 2 miles to 15.59 miles in China. The values of wind speed were between 0 knot and 19.9 knots in the 28 countries, and from 1.31 knots to 11.45 knots in China. City elevation ranged from -0.0658 hectometers to 21.0429 hectometers in the 28 countries, and from 0.05 hectometers to 22.5658 hectometers in China. The minimum and maximum values of the dew point were -21.86°F and 77.25°F in the 28 countries and 4.19°F and 76.73°F in China. Most respondents (64%) in the 28 countries experienced improving GDP. The distributions of age and gender are similar in both groups. Compared to their counterparts in other countries, more residents in China appeared to be in either fair or poor health and did not visit a doctor as often. The percentages of never smoking, never drinking, and never doing physical activity were all higher in China, where people eat fruits or vegetables more often than their counterparts in other countries.

**Table 1:** Descriptive statistics of the Macro-level explanatory variables.

Macro variables	28 countries (N = 25,441 Records)				China (N = 5,420 Records)			
	Min.	Mean	Max.	S.D.	Min.	Mean	Max.	S.D.
Temperature (°F)	-17.41	56.28	87.6	14.6	26.62	75.93	90.32	8.33
Dew point (°F)	-21.86	44.83	77.25	12.83	4.19	64.62	76.73	10.1
Visibility (miles)	0	8.98	31.76	4.16	2	6.86	15.59	2.42
Wind speed (knots)	0	6.62	19.9	2.83	1.31	4.47	11.45	1.3
City elevation (hectometers)	-0.0658	2.999	21.0429	3.4572	0.05	3.2696	22.5658	5.5104
GDP (By country)	N		%		N		%	
Decrease	9,159		36.00%		-		-	
Increase	16,282		64.00%		-		-	

'- ': not available

**Table 2:** Descriptive statistics of the micro-level explanatory variables.

Micro variables		28 countries (N = 25,441 Records)		China (N = 5,420 Records)	
		N(avg.)	% (s.d.)	N(avg.)	% (s.d.)
Gender	Male	11,446	45.04%	2447	45.15%
	Female	13,968	54.96%	2973	54.85%
Age (years)		49.15 ^a^	17.5^b^	47.78 ^a^	16.02 ^b^
Health status	Excellent	1,788	7.27%	450	8.34%
	Very good	5,674	23.06%	1296	24.03%
	Good	10,504	42.69%	1140	21.14%
	Fair	5,436	22.09%	1885	34.95%
	Poor	1,204	4.89%	622	11.53%
Past 12 months: visit a doctor	Never	4,074	16.28%	1307	24.27%
	Seldom	8,089	32.31%	1926	35.76%
	Sometimes	7,899	31.56%	1359	25.23%
	Often	3,556	14.21%	695	12.90%
	Very often	1,414	5.65%	99	1.84%
Smoking cigarettes	Do not smoke and never did	12,468	49.55%	3591	66.55%
	Do not smoke now, but smoked in the past	6,585	26.17%	351	6.50%
	Smoke 1–5 cigarettes per day	1,546	6.14%	368	6.82%
	Smoke 6–10 cigarettes per day	1,684	6.69%	332	6.15%
	Smoke 11–20 cigarettes per day	2,304	9.16%	575	10.66%
	Smoke 21–40 cigarettes per day	519	2.06%	145	2.69%
	Smoke more than 40 cigarettes per day	56	0.22%	34	0.63%
Drinks alcohol	Never	14,510	57.90%	3876	72.34%
	Once a month or less often	6,014	24.00%	704	13.14%
	Several times a month	3,123	12.46%	309	5.77%
	Several times a week	1,064	4.25%	245	4.57%
	Daily	348	1.39%	224	4.18%
Physical activity	Never	5,426	21.94%	2475	46.59%
	Once a month or less often	3,455	13.97%	825	15.53%
	Several times a month	5,291	21.39%	492	9.26%
	Several times a week	6,778	27.41%	466	8.77%
	Daily	3,781	15.29%	1054	19.84%
Eat fresh fruits or vegetables	Never	319	1.27%	59	1.10%
	Once a month or less often	716	2.85%	136	2.54%
	Several times a month	2,535	10.08%	261	4.88%
	Several times a week	7,845	31.20%	942	17.60%
	Daily	13,728	54.60%	3953	73.87%

^a^. This parameter is an average value.

^b^. This parameter is a standard deviation.

### Statistical analysis

Based on the ordinal logistic regression model, the results from the 28 countries are shown in Table 3. Most macro background factors turned out to be highly relevant with happiness in different periods. People were happier, for example, when and where temperature, visibility, and wind speed were all higher, as measured by various intervals (mostly significant at the .01 level). Except for the dew point, which had negative effects (for example, 4 Days: -0.005*; 8 Days: -0.005*), all weather conditions and GDP showed some positive effects on happiness (significant at the .001 level).

**Table 3:** The coefficients of variables in 28 countries.

Variables		Coefficients		
		2 Days	4 Days	8 Days
Temperature (°F)		0.005**	0.006***	0.007***
Dew point (°F)		-0.003•	-0.005*	-0.005*
Visibility (miles)		0.006*	0.008**	0.011***
Wind speed (knots)		0.012**	0.017***	0.024***
City elevation (hectometers)		0.014***	0.013**	0.013**
GDP (By country) (“Increase” as the reference level)	Decrease	-0.123***	-0.122***	-0.125***
Gender (“Female” as the reference level)	Male	0.07*	0.07*	0.071**
Age (years)		-0.004***	-0.004***	-0.004***
Health status (“Excellent” as the reference level)	Poor	-3.151***	-3.147***	-3.137***
	Fair	-2.116***	-2.114***	-2.11***
	Good	-1.311***	-1.308***	-1.304***
	Very good	-0.639***	-0.639***	-0.642***
Past 12 months: visit a doctor (“Very often” as the reference level)	Never	-0.081	-0.075	-0.063
	Seldom	-0.03	-0.028	-0.019
	Sometimes	-0.061	-0.056	-0.044
	Often	-0.004	-0.002	0.008
Smoking cigarettes (“Smoke more than 40 cigarettes per day” as the reference level)	Do not smoke and never did	0.225	0.227	0.235
	Do not smoke now, but smoked in the past	0.295	0.295	0.301
	Smoke 1–5 cigarettes per day	0.229	0.235	0.247
	Smoke 6–10 cigarettes per day	0.017	0.017	0.025
	Smoke 11–20 cigarettes per day	-0.022	-0.023	-0.018
	Smoke 21–40 cigarettes per day	-0.244	-0.245	-0.242
Drinks alcohol (“Daily” as the reference level)	Never	0.029	0.023	0.029
	Once a month or less often	0.002	-0.003	-0.001
	Several times a month	-0.058	-0.065	-0.066
	Several times a week	-0.191	-0.197	-0.202
Physical activity (“Daily” as the reference level)	Never	-0.108*	-0.108*	-0.107*
	Once a month or less often	-0.011	-0.007	-0.009
	Several times a month	-0.006	-0.006	-0.008
	Several times a week	-0.004	-0.003	-0.005
Eats fresh fruits or vegetables (“Daily” as the reference level)	Never	-0.414***	-0.416***	-0.412***
	Once a month or less often	-0.617***	-0.624***	-0.62***
	Several times a month	-0.379***	-0.38***	-0.38***
	Several times a week	-0.164***	-0.163***	-0.16***

Signif. codes: 0 '***' 0.001 '**' 0.01 '*' 0.05 '.' 0.1 ' ' 1

In addition to geospatial factors, some individual characteristics were also closely linked to the extent of happiness. Male and younger respondents, for example, tended to be happier (Gender: 2 Days: 0.07*; 4 Days: 0.07*; 8 Days: 0.071**; Age: 2 Days: -0.004***; 4 Days: -0.004***; 8 Days: -0.004***). Self-reported health status was very strongly linked to happiness: Compared to those in excellent shape, all others felt less happy (e.g., for the 8-day sensitivity tests, Poor: -3.137***; Fair: -2.11***; Good: -1.304***; Very good: -0.642***). Somewhat unexpectedly, such a strong association also existed between happiness and the habit of eating fresh fruits and vegetables. Those who ate fruits and vegetables daily were significantly happier than those eating fruits and vegetables less often (for the 8-day sensitivity tests, Never: -0.412***; Once a month or less often: -0.62***; Several times a month: -0.38***; Several times a week: -0.16***). Among other lifestyle characteristics, only doing physical activity played a partial role in identifying who was happier: Although the frequency of doing physical activity made little difference, those who skipped exercise altogether (never doing physical activity) were significantly less happy (2 Days: -0.108*; 4 Days: -0.108*; 8 Days: -0.107*). Other than that, the extent of happiness did not vary by the frequencies of smoking, drinking alcohol, or visiting a doctor (Table 3).

To further examine whether the geospatial effects showed any temporal or regional variations, we conducted similar analyses taking both seasons and climate zones into account (Table 4). Because the vast majority of the countries in our study are located in the northern hemisphere, the season variable needed to be adjusted only for Australia and Chile. Because the number of countries with sufficient information was limited, we combined climate zones into either Tropical/Subtropical or Temperate/Cold, with the latter being the reference group in the statistical analyses.

**Table 4:** The four seasons coefficients of variables in 28 countries.

Season	Variables		Coefficients		
			2 Days	4 Days	8 Days
Spring	Dew point (°F)		-0.004*	-0.005*	-0.004•
	Visibility (miles)		-0.002	-0.001	0.002
	Wind speed (knots)		0.011	0.014•	0.018*
	City elevation (hectometers)		0.023***	0.02***	0.023***
	City Climate Zone (“Temperate/Cold” as the reference level)	Tropical/Subtropics	-0.139**	-0.079	0.129*
Summer	Dew point (Fahrenheit)		-0.009**	-0.008*	-0.008*
	Visibility (miles)		-0.007	-0.005	-0.003
	Wind speed (knots)		0.024*	0.029**	0.032**
	City elevation (hectometers)		0.004	0.004	0.005
	City Climate Zone (“Temperate/Cold” as the reference level)	Tropical/Subtropics	-0.024	-0.031	-0.036
Autumn	Dew point (Fahrenheit)		0.003	0.004	0.005
	Visibility (miles)		0.02**	0.025***	0.034***
	Wind speed (knots)		-0.011	-0.004	0.014
	City elevation (hectometers)		-0.031*	-0.024•	-0.011
	City Climate Zone (“Temperate/Cold” as the reference level)	Tropical/Subtropics	0.174*	0.197*	0.285**
Winter	Dew point (Fahrenheit)		-0.006*	-0.009**	-0.009**
	Visibility (miles)		-0.019	-0.025•	-0.019
	Wind speed (knots)		0.008	0.02•	0.015
	City elevation (hectometers)		-0.035*	-0.025•	-0.038*
	City Climate Zone (“Temperate/Cold” as the reference level)	Tropical/Subtropics	0.687***	0.769***	0.67***

Signif. codes: 0 '***' 0.001 '**' 0.01 '*' 0.05 '.' 0.1 ' ' 1

In the spring, city elevation was positively associated with a greater level of happiness (2 Days: 0.023***; 4 Days: 0.02***; 8 Days: 0.023***), while a lower dew point also helped. Those living in tropical/subtropical zones were not as happy as their counterparts in temperate/cold zones. The effect of dew point on happiness remained significantly negative in summer, but other geospatial effects showed a very different pattern from that in spring (2 Days: -0.004*; 4 Days: -0.005*). Neither city elevation nor climate zone played a significant role in summer, but the wind speed had a significantly positive effect on happiness.

The seasonal patterns also differed between autumn and winter. Even though a higher dew point remained a negative factor in terms of residents’ happiness in winter (2 Days: -0.006*; 4 Days: -0.009**; 8 Days: -0.009**), better visibility emerged as a positive factor in autumn (2 Days: 0.02**; 4 Days: 0.025***; 8 Days: 0.034***), the only season when this particular weather condition showed a significant effect. Furthermore, unlike its positive effect in spring, higher city elevation turned out to be a negative factor for personal happiness in both autumn and winter (In autumn: 2 Days: -0.031*; In winter: 2 Days: -0.035*; 8 Days: -0.038*). While residents in tropical/subtropical zones were not as happy as those living in temperate/cold zones in spring, they became significantly happier than their counterparts from such colder climate zones when autumn arrived (2 Days: 0.174*; 4 Days: 0.197*; 8 Days: 0.285**); and this climate zone positive effect lasted into winter (2 Days: 0.687***; 4 Days: 0.769***; 8 Days: 0.67***).

Overall, both seasonal and climate zone variations in the geospatial effects on personal happiness were evident in these 28 countries. Such variations remained unknown in China, because the Chinese interviews of the ISSP module were mainly completed in the summer. Without taking season and climate zones into account, the analyses of geospatial factors and individual characteristics for China showed some results very similar to the findings from the 28 countries. For example, higher temperature (2 Days: 0.017**; 4 Days: 0.018**; 8 Days: 0.021**), lower dew point (2 Days: -0.019***; 4 Days: -0.02**; 8 Days: -0.022**), and higher city elevation (2 Days: 0.032***; 4 Days: 0.032***; 8 Days: 0.031***) were all positively associated with happiness (Table 5). As in the other countries, furthermore, being in excellent health and eating fruits or vegetables daily were both very strong indicators for being a happier person in China.

**Table 5:** The coefficients of variables in China.

Variables		Coefficients		
		2 Days	4 Days	8 Days
Temperature (°F)		0.017**	0.018**	0.021**
Dew point (°F)		-0.019***	-0.02**	-0.022**
Visibility (miles)		-0.018	-0.021	-0.017
Wind speed (knots)		0.007	-0.003	-0.044*
City elevation (hectometers)		0.032***	0.032***	0.031***
Gender (“Female” as the reference level)	Male	-0.092	-0.091	-0.091
Age (years)		0.007***	0.007***	0.007***
Health status (“Excellent” as the reference level)	Poor	-1.86***	-1.855***	-1.856***
	Fair	-1.087***	-1.082***	-1.075***
	Good	-0.893***	-0.894***	-0.894***
	Very good	-0.541***	-0.541***	-0.542***
Past 12 months: visit a doctor (“Very often” as the reference level)	Never	-0.2	-0.199	-0.202
	Seldom	-0.297	-0.3	-0.305
	Sometimes	-0.223	-0.224	-0.232
	Often	-0.197	-0.202	-0.212
Smoking cigarettes (“Smoke more than 40 cigarettes per day” as the reference level)	Do not smoke and never did	0.113	0.109	0.124
	Do not smoke now, but smoked in the past	0.131	0.128	0.146
	Smoke 1–5 cigarettes per day	0.034	0.033	0.053
	Smoke 6–10 cigarettes per day	0.024	0.019	0.038
	Smoke 11–20 cigarettes per day	-0.067	-0.068	-0.043
	Smoke 21–40 cigarettes per day	0.027	0.02	0.034
Drinks alcohol (“Daily” as the reference level)	Never	-0.181	-0.178	-0.176
	Once a month or less often	-0.343*	-0.338*	-0.343*
	Several times a month	-0.208	-0.205	-0.214
	Several times a week	-0.28	-0.278	-0.286
Physical activity (“Daily” as the reference level)	Never	-0.151*	-0.153*	-0.151*
	Once a month or less often	-0.04	-0.042	-0.037
	Several times a month	0.01	0.008	0.017
	Several times a week	0.018	0.02	0.028
Eats fresh fruits or vegetables (“Daily” as the reference level)	Never	-0.666*	-0.673*	-0.679*
	Once a month or less often	-0.671***	-0.673***	-0.67***
	Several times a month	-0.779***	-0.783***	-0.778***
	Several times a week	-0.437***	-0.441***	-0.446***

Signif. codes: 0 '***' 0.001 '**' 0.01 '*' 0.05 '.' 0.1 ' ' 1

## Discussion

The uniqueness of this study is the integration of globally representative survey data (from the ISSP 2011 module) with the corresponding weather data, geo-information about the city, and the economic development index. Few previous studies have used large samples, long temporal periods, and wide spatial coverage to elucidate the correlation between happiness and weather factors. One of the most critical and challenging tasks for data integration lies in linking such macro and micro data at the city level by a geospatial method. The daily weather data and the individual-level background factors in this study are detailed enough for examining short-term weather effects on happiness, while controlling for possible confounders among respondents. To our knowledge, this is the first paper to use both global survey data and world weather data with an advanced geoprocessing method to address the macro and micro effects on happiness.

At the macro level, several factors were found to be strongly linked to happiness. A previous study [17] showed that weather conditions, such as humidity, wind speed, precipitation, and sunshine, were not significantly associated with happiness, but temperature remained an important factor that helped distinguish happiness among people. Other research has also shown a positive correlation between the temperature in the coldest months and the degrees of happiness from the world database of happiness [1]. In addition, people tend to feel less happy as the dew point becomes higher [18]. According to yet another study, better visibility also had a significant effect on happiness [19], which can also be found from our analyses.

With our unique approach, the analyses of seasonal variations further enrich discussions on the geospatial effects on happiness. It appears that in the spring, people enjoy less humid weather and higher elevation. It is also interesting to note that those living in temperate and cold zones are happier than those living in tropical and subtropical zones. It is reasonable to argue that when spring arrives, residents in temperate and cold zones experience a more drastic temperature change from cold to warm weather than those living in tropical and subtropical zones, which often brings about some excitement and surprises in everyday life. In contrast, people living in tropical and subtropical zones are happier than their counterparts in temperate and cold zones in both autumn and winter. The reason is similar: Residents in temperate and cold zones feel a larger temperature drop from warm to cold than those living in tropical and subtropical zones. Also in both autumn and winter, higher elevations make it colder than lower ones, which in turn impedes short-term subjective happiness. In short, the incorporation of both season and climate zone into the models further enriches our geospatial approach to studying personal happiness.

In addition to geospatial circumstances like weather conditions, a societal factor, GDP, also exerted a significantly positive effect on happiness among residents in the 28 countries. Some previous studies have shown a similar positive and significant association between growth in GDP per capita and happiness. Hagerty and Veenhoven [20], for example, found that GDP was positively related to the number of “happy life years” in 14 of the 21 countries available in their dataset. In a later study, Hagerty and Veenhoven [21] also observed a statistically significant rise in happiness in 4 out of 8 high-income countries and 3 out of 4 low-income countries. In light of these recent studies, the current paper reconfirms the positive impact of an increase in GDP on personal happiness, while taking into account both geospatial factors and individual characteristics from two large-scale global datasets.

At the individual level, whether in 28 countries or in China, two of the personal characteristics and lifestyles, health status and the frequency of eating fresh fruits and vegetable, were consistently linked to happiness. Health and well-being were always interconnected, with well-being influencing health and health influencing well-being [22, 23]. Good health was linked with greater happiness, while setbacks in health, such as serious diseases or disability, had negative effects on happiness. Likewise, it is widely known that eating fresh fruits and vegetables is good for your health. Happiness represents a relatively new field of study that is nonetheless in great demand today; but surprisingly, scarce attention has been given to people’s eating habits in happiness studies. A recent study did conclude that a person’s mental well-being might be associated with the consumption of fruits [24]. With very similar research results about this lifestyle effect, while also controlling for weather conditions and societal development, our geospatial approach of happiness studies should further strengthen the significance of this particular habit of food consumption.

Our geospatial approach also confirmed the linkage between exercise and happiness. Respondents with no physical activity tended to experience significantly a lower happiness level than those doing physical activity, regardless of the frequency. Similarly, a multi-country analysis of the association between physical activity and happiness found that those who engaged in daily physical activity were more likely to be happy [25]. Some previous studies have found that unhealthy habits, such as smoking, drinking alcohol, and not exercising, were closely linked to substantially lower happiness scores [24–26]. Although our study confirmed a linkage between happiness and physical activity, neither smoking nor drinking alcohol showed any significant, consistent relationship with happiness.

Compared to the overall findings from the global dataset, the analyses of the Chinese data unveiled some interesting and contrasting results. Happiness, for example, was clearly linked with gender in the 28 countries, where men were happier than women, but such a gender difference was lacking in China. While younger people were happier than older people in the 28 countries, older people were happier than their younger counterparts in China.

According to relevant research, such an age effect may be due to cognitive processes, particularly the processes that focus on and are related to remembering positive events, while leaving behind negative ones. If such processes are indeed at work, they may help older people regulate their emotions, letting them view life in a sunnier light [27, 28]. In western culture, being happy is seen more as a personal accomplishment. Under the influence of Confucianism, however, Chinese people often regard interpersonal relationships as a key factor in defining happiness [29]. As a result, women in China may feel happier if they fulfill their role obligations in a family, which in turn helps ensure family harmony, as well as prosperity. By contrast, men in China tend to rely on social status and material achievement to a greater extent for their happiness [30].

Due to the nature of the approach and the data collected at different levels in different countries and regions, however, several limitations and concerns deserve discussion in more details. First, the ISSP survey data do not cover residents in the tropical zone, which presents some representativeness problems if we want to discuss happiness in that climate zone individually. Second, because the response scales of happiness differ between the 28 countries and China, we could not compile both survey datasets together. The split of the subsamples may have slightly reduced the power for explaining happiness on a global level. As an alternative, we treated the China data as our validation data. Third, we used the average weather variables within each city. Because we did not know the exact location of every respondent, we could only average the weather factors from all the stations within a city. This rough estimation may ignore the variability of weather conditions within a city.

Fourth, social phenomena involving macro- and micro-level data can be better understood with countries’ or regions’ dummy explanatory variables, or using multilevel analyses. Because the survey data were collected from individuals, while the weather data were aggregate statistics of the cities or areas where a number of these survey respondents lived, statistical analyses of personal happiness should take into account how the repeated measures of the weather data may affect the variation in personal happiness. The GDP was also aggregate data at the country level. When we tried incorporating countries or regions as dummy variables, part of the regression outcomes became somewhat unstable. The distribution of the 29 countries used in this study is scarce and uneven compared with the total number of countries in different geographical regions, as categorized by United Nations (S3 Table). Except for Eastern and Western Europe, most geographical regions are under-represented. Further analyses controlling for countries or regions would be more feasible and reliable in the future when survey data from more countries become available.

Fifth, data compatibility remains a major issue in comparative studies, especially when the comparisons involve subjective survey items. The ordering and wording of question items and response categories, for example, need to be identical in different surveys for the data to be compatible. Furthermore, sampling and interviewing need to be designed and administered in the manner that data come from samples that well represent the populations. Any incoherent designs and practices may cause potential biases that no advanced statistical analyses can amend. Such biases may be particularly serious when comparing survey data across different countries and cultures, which requires the continued invariant measurement of constructs [31]. Even when question items are well translated in different countries, respondents from various cultural backgrounds may interpret them in a manner that deviates from the original design. As a result, the differences found across countries could be methodological artifacts rather than real differences. To prevent discovery of such artifacts, it is critical to design and follow specific precautions in all survey stages to assess and ensure data comparability.

The global survey dataset we used, the 2010 ISSP module, was available through GESIS. Started from 1984, the ISSP has been continuously conducting annual surveys on diverse topics, covering various cultures around the globe [12, 32]. To achieve data comparability, the scientific committees of the ISSP compose and revise detailed project specifications for each successive round of survey. All member teams are required to conduct and document fieldwork comprehensively according to the same standard setups. Such a principle of equality applies to sample selection, translation of the questionnaire, and all methods and processes associated with data collection and processing [12]. With such rigid requirements, the ISSP ensures compatibility across countries and cultures in questionnaire design, sampling, fielding, and data processing.

For data processing in the current study, we filtered out incomplete and unreasonable cases to extract useful information for geocoding. To adjust for variations in the dates of interviews, we further used dummy variables for season, climate zone, and GDP. Combined with the high quality of the ISSP data, such solutions in data process should have enhanced data comparability to the extent that each ISSP country made their best efforts in complying with the standardized setups.

## Conclusions

The current study has taken advantage of widely available global datasets that have emerged and blossomed in recent years. While world-wide survey and weather data have facilitated various studies and inspired research insights, our study moved a step further by combining individual-level survey data with city-level weather data. The research findings based on the geospatial approach shed new light on the substantive research subject.

The factors linked to personal happiness are quite complex. This study tried to integrate international survey data with global weather data by a geospatial approach. With a macro perspective, the ordinal logistic analyses indicated that people living under the conditions of higher temperature, higher visibility, a little wind, lower dew point, and an improving economic situation felt happier. In an effort to distinguish the effects of weather conditions on happiness in different seasons and different climate zones, further analyses revealed significant seasonal variations in these geospatial effects. At the individual level, the same ordinal logistic analyses reconfirmed that better health condition and eating more fruits or vegetables were both highly associated with happiness, while taking into account geospatial factors and societal development. Never doing physical activity appeared to make people less happy. The findings from these macro- and micro-level background factors suggest that weather conditions, economic situations, and personal health behaviors all provide a better understanding of subjective happiness at the individual level. Adding the geospatial approach to the regression analyses of social survey data thus helps broaden and enhance happiness studies.

## Supporting Information

S1 File The model diagnostic results. (PDF)

S1 Table List of ISSP member countries of the 2010 data. (PDF)

S2 Table The SPSS syntax used for ordinal logistic regression. (PDF)

S3 Table Number of countries in this study and in the UN data. (PDF)
