# Supplementary material for: Analyzing Personal Happiness from Global Survey and Weather Data: A Geospatial Approach
Source: PLoS One. 2016 Apr 14;11(4):e0153638. doi: 10.1371/journal.pone.0153638 (PMC4831775; doi:10.1371/journal.pone.0153638)
Supplement: S1 File — (PDF) [file pone.0153638.s001.pdf]

**S1 File. The model diagnostic results**

The model diagnostics are methods for determining whether a fitted model adequately represents the data. To evaluate the ordinal regression models in this study, we need to determine whether the model improves our ability to explain the outcome. We do this by comparing the ordinal regression model without any explanatory variables (the "Intercept Only" model) against the model with all the explanatory variables (the "Final" model). We compare the Final model against the Intercept Only model to see whether it significantly improves the fit to the data. S1 File Tables 1 and 2 show the model fitting information. The statistically significant chi-square statistics indicate that the Final models are a significant improvement over the Intercept Only models.

A standard statistical maneuver for testing whether a model fits is to compare the observed data with the fitted model for consistency. From the observed and expected frequencies, the usual Pearson and Deviance goodness-of-fit measures are computed in S1 File Tables 3 and 4. We start from the null hypothesis that the fit is good. If we do not reject this hypothesis (i.e., if the p value is large), then we conclude that the observed data and the model predictions are similar and that we have a good model. The results for our analysis suggest all models in this study fitted well ($p>0.01$).

To assess the strength of association, there are several $R^2$-like statistics that can be used to measure the strength of the association between the response variable and the explanatory variables. In this study, three commonly used pseudo $R^2$ statistics are employed to measure the strength of association. The results are shown in S1 File Tables 5 and 6.

Here, the Nagelkerke's pseudo $R^2$ values (16.80% for the 28 countries' data and 8.80% for the China data) indicate that the model explains a relatively small proportion of the variation between people in their happiness levels. This is just as we would expect, because there are numerous factors that affect personal happiness.

Multicollinearity occurs when statistical models have two or more explanatory variables that are highly correlated with each other. This leads to problems with understanding which variable contributes to the explanation of the response variable and technical issues in calculating an ordinal regression. Determining whether there is multicollinearity is an important step in ordinal regression. To test for this assumption, we require creating dummy variables for each level of the categorical explanatory variables in this study. We use the Variance Inflation Factor (VIF) value for each

variable as a check for multicollinearity, and as a rule of thumb, we use the VIF of an explanatory variable greater than 10 as a cutoff for variables that may merit further investigation. The results in S1 File Tables 7 and 8 indicate that the VIF values are all quite acceptable for the significant variables in this study. Two variables, “smoking cigarettes” and “past 12 months: have visited a doctor,” have large VIF values. Both of them, however, are non-significant in our models.

The key assumption for fitting an ordinal regression is that the effects of any explanatory variables are consistent or proportional across different thresholds; hence this is usually termed the assumption of proportional odds (*S*PSS calls this the assumption of parallel lines). We evaluate the appropriateness of this assumption through “the test of parallel lines” in SPSS. This test compares the ordinal model, which has one set of coefficients for all thresholds (labelled Null Hypothesis), to a model with a separate set of coefficients for each threshold (labelled General). The test results in S1 File Tables 9 and 10 give a significantly better fit to the two datasets than the ordinal model, and thus we were led to reject the proportional odds assumption. The test of the proportional odds assumption has been described as anti-conservative, however, in that it nearly always results in rejection of the proportional odds assumption [1], particularly when the number of explanatory variables is large [2], the sample size is large [3, 4], or there is a continuous explanatory variable in the model [3]. It is important to examine the data using multinomial logistic regression to explicitly see how the odds ratios (ORs) for our explanatory variables vary at the different thresholds. We use the 2-day period ordinal regression for 28 countries as an illustration. Looking at the separate ORs of continuous explanatory variables across the six splits in S1 File Table 11, the difference in ORs appears negligible (0.963 to 1.005 for *Age*), so a common OR for each of these continuous explanatory variables is a very plausible assumption. The proportional odds assumption is also upheld for most of the categorical variables.

The categorical variable most out of line with the proportional odds assumption is smoking cigarettes. Of 25,096 respondents to this question, 12,432 (49.50%) replied, “Do not smoke and never did”; 6,567 (26.25%) replied, “Do not smoke now but smoked in the past”; 1,544 (6.20%) replied, “Smoke 1-5 cigarettes per day”; 1,683 (6.70%) replied, “Smoke 6-10 cigarettes per day”; 2,296 (9.10%) replied, “Smoke 11-20 cigarettes per day”; 518 (2.10%) replied, “Smoke 21-40 cigarettes per day”; and 56 (0.20%) replied, “Smoke more than 40 cigarettes per day.” The ORs for smoking cigarettes from the separate logistic regressions differ hugely. In this particular case, it might be reasonable to conclude that the ORs from the ordinal

regression model do underestimate the extent of the over-representation at the "Do not smoke and never did" level, the "Do not smoke now but smoked in the past" level, the "Smoke 6-10 cigarettes per day" level, and the "Smoke 11-20 cigarettes per day" level, and do overestimate the extent of the under-representation at the "Smoke 1-5 cigarettes per day" level and the "Smoke 21-40 cigarettes per day" level. This finding was obscured in the single cumulative OR for each level of smoking cigarettes, and summarizing this relationship in a single OR misses this observation. Thus, smoking cigarettes may well be the major factor underlying the overall rejection of parallel lines. The Chi-square test that led to the rejection of the proportional odds assumption probably reflects the large sample size in our datasets. We think the violation of the proportional odds assumption is quite minor.

References:

[1] O'Connell, A. (2006). Logistic regression models for ordinal response variables. Thousand Oaks: Sage Publications.

[2] Brant, R. (1990). Assessing proportionality in the proportional odds model for ordinal logistic regression. Biometrics, 46, 1171-1178.

[3] Allison, P. D. (1999). Logistic regression using the SAS system: Theory and application. Cary, NC.: SAS Institute.

[4] Clogg, C., & Shihadeh, E. S. (1994). Statistical models for ordinal variables. Thousand Oaks, California: Sage Publications.

S1 File Table 1. Model fitting information for ordinal regressions of 28 countries' data

Model Fitting Information

| Model | | Intercept Only | Final |
|---|---|---|---|
| -2 Log Likelihood | 2 Days | 61923.621 | 58188.923 |
| | 4 Days | 62036.285 | 58279.698 |
| | 8 Days | 62146.090 | 58373.945 |
| Chi-Square | 2 Days | | 3734.699 |
| | 4 Days | | 3756.587 |
| | 8 Days | | 3772.145 |
| df | 2 Days | | 34 |
| | 4 Days | | 34 |
| | 8 Days | | 34 |
| Sig. | 2 Days | | 0.000 |
| | 4 Days | | 0.000 |
| | 8 Days | | 0.000 |

S1 File Table 2. Model fitting information for ordinal regressions of China data

Model Fitting Information

| Model | | Intercept Only | Final |
|---|---|---|---|
| -2 Log Likelihood | 2 Days | 11737.973 | 11308.496 |
| | 4 Days | 11737.973 | 11309.800 |
| | 8 Days | 11737.973 | 11306.994 |
| Chi-Square | 2 Days | | 429.476 |
| | 4 Days | | 428.173 |
| | 8 Days | | 430.979 |
| df | 2 Days | | 33 |
| | 4 Days | | 33 |
| | 8 Days | | 33 |
| Sig. | 2 Days | | 0.000 |
| | 4 Days | | 0.000 |
| | 8 Days | | 0.000 |

S1 File Table 3. Goodness of fit for ordinal regressions of 28 countries' data

Goodness-of-Fit

| Model | | Pearson | Deviance |
|---|---|---|---|
| Chi-Square | 2 Days | 131907.774 | 58170.901 |
| | 4 Days | 132316.281 | 58261.677 |
| | 8 Days | 132543.978 | 58355.923 |
| df | 2 Days | 130964 | 130964 |
| | 4 Days | 131228 | 131228 |
| | 8 Days | 131438 | 131438 |
| Sig. | 2 Days | 0.033 | 1.000 |
| | 4 Days | 0.017 | 1.000 |
| | 8 Days | 0.016 | 1.000 |

## S1 File Table 4. Goodness of fit for ordinal regressions of China data

Goodness-of-Fit

| Model | | Pearson | Deviance |
|---|---|---|---|
| Chi-Square | 2 Days | 21090.711 | 11301.565 |
| | 4 Days | 21127.919 | 11302.868 |
| | 8 Days | 21075.581 | 11300.063 |
| df | 2 Days | 20731 | 20731 |
| | 4 Days | 20731 | 20731 |
| | 8 Days | 20731 | 20731 |
| Sig. | 2 Days | 0.039 | 1.000 |
| | 4 Days | 0.026 | 1.000 |
| | 8 Days | 0.046 | 1.000 |

## S1 File Table 5. The Pseudo R-Square of 28 countries' data

Pseudo R-Square

| | 2 Days | 4 Days | 8 Days |
|---|---|---|---|
| Cox and Snell | 0.157 | 0.158 | 0.158 |
| Nagelkerke | 0.167 | 0.168 | 0.168 |
| McFadden | 0.060 | 0.061 | 0.061 |

## S1 File Table 6. The Pseudo R-Square of China data

Pseudo R-Square

| | 2 Days | 4 Days | 8 Days |
|---|---|---|---|
| Cox and Snell | 0.079 | 0.079 | 0.080 |
| Nagelkerke | 0.088 | 0.088 | 0.089 |
| McFadden | 0.037 | 0.036 | 0.037 |

S1 File Table 7. VIF values of variables for diagnosing multicollinearity of 28 countries' data

Coefficients[a]

| Model | | Collinearity Statistics | | |
|---|---|---|---|---|
| | | VIF | | |
| | | 2 Days | 4 Days | 8 Days |
| | (Constant) | | | |
| | Q26 Health status (Poor) | 1.646 | 1.647 | 1.648 |
| | Q26 Health status (Fair) | 2.785 | 2.788 | 2.791 |
| | Q26 Health status (Good) | 3.127 | 3.136 | 3.143 |
| | Q26 Health status (Very good) | 2.494 | 2.499 | 2.505 |
| | Q18a Past 12 months: visit a doctor (Never) | 3.066 | 3.064 | 3.065 |
| | Q18a Past 12 months: visit a doctor (Seldom) | 4.174 | 4.172 | 4.173 |
| | Q18a Past 12 months: visit a doctor (Sometimes) | 3.965 | 3.965 | 3.967 |
| | Q18a Past 12 months: visit a doctor (Often) | 2.604 | 2.602 | 2.601 |
| | Q24 Smoking cigarettes (Do not smoke and never did) | 20.257 | 20.293 | 20.327 |
| | Q24 Smoking cigarettes (Do not smoke now but smoked in the past) | 15.692 | 15.712 | 15.745 |
| | Q24 Smoking cigarettes (Smoke 1-5 cigarettes per day) | 5.534 | 5.540 | 5.545 |
| | Q24 Smoking cigarettes (Smoke 6-10 cigarettes per day) | 5.895 | 5.898 | 5.899 |
| | Q24 Smoking cigarettes (Smoke 11-20 cigarettes per day) | 7.439 | 7.450 | 7.465 |
| | Q24 Smoking cigarettes (Smoke 21-40 cigarettes per day) | 2.547 | 2.547 | 2.550 |
| | Q25a How often: drink alcohol (Never) | 9.690 | 9.692 | 9.696 |
| | Q25a How often: drink alcohol (Once a month or less often) | 7.589 | 7.586 | 7.586 |
| | Q25a How often: drink alcohol (Several times a month) | 4.908 | 4.912 | 4.915 |
| | Q25a How often: drink alcohol (Several times a week) | 2.397 | 2.402 | 2.402 |
| | Q25b How often: physical activity (Never) | 1.890 | 1.891 | 1.892 |
| | Q25b How often: physical activity (Once a month or less often) | 1.632 | 1.632 | 1.632 |
| | Q25b How often: physical activity (Several times a month) | 1.827 | 1.826 | 1.826 |
| | Q25b How often: physical activity (Several times a week) | 1.920 | 1.920 | 1.920 |
| | Q25c How often: eat fresh fruits or vegetables (Never) | 1.029 | 1.029 | 1.029 |
| | Q25c How often: eat fresh fruits or vegetables (Once a month or less | 1.059 | 1.059 | 1.058 |
| | Q25c How often: eat fresh fruits or vegetables (Several times a month) | 1.146 | 1.145 | 1.146 |
| | Q25c How often: eat fresh fruits or vegetables (Several times a week) | 1.150 | 1.150 | 1.151 |
| | Sex of Respondent (Male) | 1.166 | 1.166 | 1.167 |
| | GDP (Decrease) | 1.097 | 1.102 | 1.109 |
| | TEMP | 3.897 | 4.045 | 4.292 |
| | DEWP | 3.791 | 3.924 | 4.181 |
| | VISIB | 1.099 | 1.110 | 1.113 |
| | WDSP | 1.079 | 1.094 | 1.103 |
| | city_z_new | 1.214 | 1.231 | 1.255 |
| | Age of respondent | 1.255 | 1.256 | 1.256 |

a. Dependent Variable: Q1 How happy or unhappy

S1 File Table 8. VIF values of variables for diagnosing multicollinearity of China data

Coefficients[a]

| Model | Collinearity Statistics VIF 2 Days | 4 Days | 8 Days |
|---|---|---|---|
| (Constant) | | | |
| Q26 Health status (Poor) | 2.442 | 2.446 | 2.446 |
| Q26 Health status (Fair) | 3.538 | 3.541 | 3.544 |
| Q26 Health status (Good) | 2.791 | 2.792 | 2.791 |
| Q26 Health status (Very good) | 2.889 | 2.889 | 2.889 |
| Q18a Past 12 months: visit a doctor (Never) | 8.812 | 8.817 | 8.818 |
| Q18a Past 12 months: visit a doctor (Seldom) | 10.640 | 10.641 | 10.641 |
| Q18a Past 12 months: visit a doctor (Sometimes) | 8.848 | 8.849 | 8.850 |
| Q18a Past 12 months: visit a doctor (Often) | 5.554 | 5.554 | 5.554 |
| Q24 Smoking cigarettes (Do not smoke and never did) | 22.460 | 22.455 | 22.444 |
| Q24 Smoking cigarettes (Do not smoke now but smoked in the | 6.730 | 6.729 | 6.729 |
| Q24 Smoking cigarettes (Smoke 1-5 cigarettes per day) | 6.978 | 6.977 | 6.976 |
| Q24 Smoking cigarettes (Smoke 6-10 cigarettes per day) | 6.441 | 6.439 | 6.438 |
| Q24 Smoking cigarettes (Smoke 11-20 cigarettes per day) | 9.953 | 9.952 | 9.950 |
| Q24 Smoking cigarettes (Smoke 21-40 cigarettes per day) | 3.462 | 3.461 | 3.461 |
| Q25a How often: drink alcohol (Never) | 4.732 | 4.732 | 4.733 |
| Q25a How often: drink alcohol (Once a month or less often) | 3.173 | 3.172 | 3.173 |
| Q25a How often: drink alcohol (Several times a month) | 2.041 | 2.041 | 2.042 |
| Q25a How often: drink alcohol (Several times a week) | 1.809 | 1.810 | 1.810 |
| Q25b How often: physical activity (Never) | 1.801 | 1.801 | 1.801 |
| Q25b How often: physical activity (Once a month or less often) | 1.528 | 1.528 | 1.529 |
| Q25b How often: physical activity (Several times a month) | 1.360 | 1.360 | 1.361 |
| Q25b How often: physical activity (Several times a week) | 1.313 | 1.313 | 1.313 |
| Q25c How often: eat fresh fruits or vegetables (Never) | 1.017 | 1.017 | 1.017 |
| Q25c How often: eat fresh fruits or vegetables (Once a month or | 1.036 | 1.036 | 1.036 |
| Q25c How often: eat fresh fruits or vegetables (Several times a | 1.044 | 1.046 | 1.048 |
| Q25c How often: eat fresh fruits or vegetables (Several times a | 1.063 | 1.065 | 1.066 |
| Sex of Respondent (Male) | 1.871 | 1.872 | 1.871 |
| TEMP | 3.808 | 4.378 | 5.510 |
| DEWP | 4.168 | 4.712 | 5.730 |
| VISIB | 1.471 | 1.442 | 1.327 |
| WDSP | 1.073 | 1.071 | 1.096 |
| city_z_new | 1.376 | 1.409 | 1.393 |
| Age of respondent | 1.213 | 1.214 | 1.214 |

a. Dependent Variable: Q1 How happy or unhappy

S1 File Table 9. Tests of parallel lines for ordinal regressions of 28 countries' data

**Test of Parallel Lines**[a]

| | | Null Hypothesis | General |
|---|---|---|---|
| -2 Log Likelihood | 2 Days | 58188.923 | 57369.177[b] |
| | 4 Days | 58279.698 | 57447.346[b] |
| | 8 Days | 58373.945 | 57530.960[b] |
| Chi-Square | 2 Days | | 819.746[c] |
| | 4 Days | | 832.352[c] |
| | 8 Days | | 842.985[c] |
| df | 2 Days | | 170 |
| | 4 Days | | 170 |
| | 8 Days | | 170 |
| Sig. | 2 Days | | 0.000 |
| | 4 Days | | 0.000 |
| | 8 Days | | 0.000 |

S1 File Table 10. Tests of parallel lines for ordinal regressions of China data

**Test of Parallel Lines**[a]

| | | Null Hypothesis | General |
|---|---|---|---|
| -2 Log Likelihood | 2 Days | 11308.496 | 11089.825[b] |
| | 4 Days | 11309.800 | 11094.175[b] |
| | 8 Days | 11306.994 | 11078.619[b] |
| Chi-Square | 2 Days | | 218.672[c] |
| | 4 Days | | 215.625[c] |
| | 8 Days | | 228.375[c] |
| df | 2 Days | | 99 |
| | 4 Days | | 99 |
| | 8 Days | | 99 |
| Sig. | 2 Days | | 0.000 |
| | 4 Days | | 0.000 |
| | 8 Days | | 0.000 |

The null hypothesis states that the location parameters (slope coefficients) are the same across response categories.[a]

a. Link function: Logit.

b. The log-likelihood value cannot be further increased after maximum number of step-halving

c. The Chi-Square statistic is computed based on the log-likelihood value of the last iteration of the general model. Validity of the test is uncertain.

# S1 File Table 11. Results of multinomial logit regression for personal happiness

| | Coefficents | | | | | | Odds Ratios | | | | | |
|---|---|---|---|---|---|---|---|---|---|---|---|---|
| Q1 How happy or unhappy | Completely unhappy | Very unhappy | Fairly unhappy | Neither happy nor unhappy | Fairly happy | Very happy | Completely unhappy | Very unhappy | Fairly unhappy | Neither happy nor unhappy | Fairly happy | Very happy |
| Intercept | -287.101 | -302.764 | 266.606 | -14.704 | -104.700 | 80.871 | - | - | - | - | - | - |
| WDSP | -.038 | .003 | -.028 | -.005 | -.016 | .005 | .963 | 1.003 | .972 | .995 | .984 | 1.005 |
| VISIB | -.048 | -.001 | .004 | -.006 | .010 | .008 | .953 | .999 | 1.004 | .994 | 1.010 | 1.008 |
| TEMP | .013 | -.011 | .001 | .001 | .010 | .013 | 1.013 | .989 | 1.001 | 1.001 | 1.010 | 1.013 |
| DEWP | -.021 | .011 | -.004 | -.006 | -.012 | -.015 | .979 | 1.011 | .996 | .994 | .988 | .985 |
| AGE | -.002 | -.003 | .004 | .006 | .007 | -.001 | .998 | .997 | 1.004 | 1.006 | 1.007 | .999 |
| city_z_new | -.040 | -.006 | -.025 | -.037 | -.031 | -.024 | .961 | .994 | .975 | .963 | .970 | .976 |
| Past 12 months: visit a doctor (Never) | -1.134 | -.213 | .054 | .386 | .377 | .164 | .322 | .808 | 1.056 | 1.471 | 1.458 | 1.178 |
| Past 12 months: visit a doctor (Seldom) | -.944 | -.351 | .260 | .492 | .546 | .447 | .389 | .704 | 1.297 | 1.636 | 1.726 | 1.563 |
| Past 12 months: visit a doctor (Sometimes) | -.640 | -.334 | .221 | .508 | .478 | .361 | .527 | .716 | 1.247 | 1.662 | 1.612 | 1.435 |
| Past 12 months: visit a doctor (Often) | -1.287 | -.687 | -.024 | .271 | .109 | .046 | .276 | .503 | .976 | 1.311 | 1.116 | 1.048 |
| [V42=5] | | | | | | | | | | | | |
| Smoking cigarettes (Do not smoke and never did) | 282.679 | 299.390 | -270.313 | 12.362 | 103.300 | -81.587 | 5.8348E+122 | 1.055E+130 | 4.025E-118 | 233830.388 | 7.2916E+44 | 3.691E-36 |
| Smoking cigarettes (Do not smoke now but smoked in the past) | 282.234 | 299.209 | -270.462 | 12.347 | 103.316 | -81.546 | 3.7397E+122 | 8.809E+129 | 3.464E-118 | 230257.621 | 7.4043E+44 | 3.845E-36 |
| Smoking cigarettes (Smoke 1-5 cigarettes per day) | 282.895 | 299.161 | -270.359 | 12.381 | 103.174 | -81.641 | 7.238E+122 | 8.395E+129 | 3.840E-118 | 238280.289 | 6.4224E+44 | 3.499E-36 |
| Smoking cigarettes (Smoke 6-10 cigarettes per day) | 283.129 | 299.372 | -269.972 | 12.448 | 103.198 | -81.815 | 9.1479E+122 | 1.037E+130 | 5.656E-118 | 254632.337 | 6.5816E+44 | 2.938E-36 |
| Smoking cigarettes (Smoke 11-20 cigarettes per day) | 282.853 | 299.356 | -269.917 | 12.756 | 103.549 | -81.567 | 6.9414E+122 | 1.02E+130 | 5.977E-118 | 346569.468 | 9.3471E+44 | 3.768E-36 |
| Smoking cigarettes (Smoke 21-40 cigarettes per day) | 283.992 | 300.464 | -269.443 | 12.620 | 103.013 | -81.870 | 2.1682E+123 | 3.09E+130 | 9.605E-118 | 302399.364 | 5.4695E+44 | 2.783E-36 |
| [V55=7] | | | | | | | | | | | | |
| How often: drink alcohol (Never) | 1.174 | -.480 | -.062 | .054 | -.145 | .018 | 3.236 | .619 | .940 | 1.055 | .865 | 1.018 |
| How often: drink alcohol (Once a month or less often) | .720 | -.223 | .182 | .104 | .097 | .154 | 2.055 | .800 | 1.200 | 1.110 | 1.102 | 1.166 |
| How often: drink alcohol (Several times a month) | .942 | -.107 | .235 | .239 | .231 | .215 | 2.564 | .898 | 1.265 | 1.270 | 1.260 | 1.240 |
| How often: drink alcohol (Several times a week) | .730 | .343 | .646 | .325 | .202 | .143 | 2.076 | 1.409 | 1.907 | 1.384 | 1.224 | 1.154 |
| [V56=5] | | | | | | | | | | | | |
| How often: physical activity (Never) | .257 | -.074 | .127 | .145 | -.045 | -.057 | 1.292 | .929 | 1.135 | 1.156 | .956 | .944 |
| How often: physical activity (Once a month or less often) | -.330 | -.302 | .101 | .224 | .324 | .194 | .719 | .740 | 1.106 | 1.251 | 1.383 | 1.214 |
| How often: physical activity (Several times a month) | -.473 | -.094 | .138 | .153 | .255 | .186 | .623 | .911 | 1.149 | 1.166 | 1.290 | 1.205 |
| How often: physical activity (Several times a week) | -.238 | -.105 | .287 | .068 | .239 | .176 | .788 | .901 | 1.333 | 1.071 | 1.269 | 1.192 |
| [V57=5] | | | | | | | | | | | | |
| How often: eat fresh fruits or vegetables (Never) | 4.944 | 1.001 | .445 | .458 | .238 | .040 | 140.375 | 2.720 | 1.560 | 1.582 | 1.269 | 1.040 |
| How often: eat fresh fruits or vegetables (Once a month or less often) | 1.908 | 1.661 | 1.629 | .331 | -.047 | -.081 | 6.741 | 5.264 | 5.097 | 1.393 | .954 | .923 |
| How often: eat fresh fruits or vegetables (Several times a month) | .118 | .824 | 1.042 | .408 | .100 | .033 | 1.125 | 2.279 | 2.835 | 1.503 | 1.105 | 1.034 |
| How often: eat fresh fruits or vegetables (Several times a week) | .098 | .283 | .415 | .261 | .120 | .069 | 1.103 | 1.327 | 1.514 | 1.299 | 1.127 | 1.071 |
| [V58=5] | | | | | | | | | | | | |
| Health status (Poor) | 9.178 | 8.217 | 6.541 | 3.871 | 2.851 | 1.504 | 9679.177 | 3702.193 | 693.154 | 48.012 | 17.298 | 4.499 |
| Health status (Fair) | 3.239 | 3.791 | 3.813 | 3.586 | 2.803 | 1.450 | 25.512 | 44.305 | 45.305 | 36.073 | 16.495 | 4.264 |
| Health status (good) | 2.311 | 2.322 | 2.510 | 2.549 | 2.645 | 1.470 | 10.086 | 10.198 | 12.302 | 12.798 | 14.079 | 4.347 |
| Health status (Very good) | 1.818 | 1.552 | 1.715 | 1.680 | 1.983 | 1.567 | 6.161 | 4.723 | 5.558 | 5.365 | 7.267 | 4.790 |
| [V59=5] | | | | | | | | | | | | |
| Sex of Respondent (Male) | -.185 | -.194 | -.218 | -.119 | -.088 | -.073 | .832 | .824 | .804 | .888 | .916 | .929 |
| [SEX=2] | | | | | | | | | | | | |
| GDP (Decrease) | -.135 | -.020 | .047 | .227 | .126 | -.011 | .874 | .981 | 1.048 | 1.255 | 1.134 | .989 |
| [GDP=1.0] | | | | | | | | | | | | |
