# Supplementary material for: Analyzing Personal Happiness from Global Survey and Weather Data: A Geospatial Approach
Source: PLoS One. 2016 Apr 14;11(4):e0153638. doi: 10.1371/journal.pone.0153638 (PMC4831775; doi:10.1371/journal.pone.0153638)
Supplement: S1 Table — (PDF) [file pone.0153638.s002.pdf]

S1 Table. List of ISSP member countries of the 2010 data

(A) **The ISSP member countries included in the current study: Australia, Belgium, Bulgaria, Chile, China, Croatia, Czech Republic, Denmark, Finland, France, Germany, Israel, Italy, South Korea, Lithuania, Netherlands, Philippines, Poland, Portugal, Russia, Slovak Republic, Slovenia, Spain, Sweden, Switzerland, Taiwan, Turkey, Great Britain, United States.**

(B) **Countries excluded from the current study: Japan, Norway, South Africa.**
