# Supplementary material for: Analyzing Personal Happiness from Global Survey and Weather Data: A Geospatial Approach
Source: PLoS One. 2016 Apr 14;11(4):e0153638. doi: 10.1371/journal.pone.0153638 (PMC4831775; doi:10.1371/journal.pone.0153638)
Supplement: S2 Table — (PDF) [file pone.0153638.s003.pdf]

S2 Table. The SPSS syntax used for ordinal logistic regression

The syntax used in 28 countries is shown below:

```
GET
  FILE='L:\HAPPY_SPSS\01_W_8days.sav'.
DATASET NAME DataSet1 WINDOW=FRONT.
PLUM V5 BY V59 V42 V55 V56 V57 V58 SEX GDP WITH TEMP DEWP VISIB WDSP city_z_new AGE
  /CRITERIA=CIN(95) DELTA(0) LCONVERGE(0) MXITER(100) MXSTEP(5) PCONVERGE(1.0E-6) SINGULAR(1.0E-8)
  /LINK=LOGIT
  /PRINT=FIT PARAMETER SUMMARY.
```

The syntax used in China is shown below:

```
GET
  FILE='L:\HAPPY_SPSS\04_C_8days.sav'.
DATASET NAME DataSet1 WINDOW=FRONT.
PLUM CN_V5 BY V59 V42 V55 V56 V57 V58 SEX WITH TEMP DEWP VISIB WDSP city_z_new AGE
  /CRITERIA=CIN(95) DELTA(0) LCONVERGE(0) MXITER(100) MXSTEP(5) PCONVERGE(1.0E-6) SINGULAR(1.0E-8)
  /LINK=LOGIT
  /PRINT=FIT PARAMETER SUMMARY.
```

The meaning of the related variables listed in the following S2 Table 1.

S2 Table 1. The variables in the query strings

Variables	Description
V5	How happy or unhappy
CN_V5	How happy or unhappy (In China)
V59	Health status
V42	Past 12 months: visit a doctor
V55	Smoking cigarettes
V56	Drinks alcohol
V57	Physical activity
V58	Eats fresh fruits or vegetables
SEX	Gender
GDP	Gross Domestic Product
TEMP	Temperature (℉)
DEWP	Dew point (℉)
VISIB	Visibility (miles)
WDSP	Wind speed (knots)
city_z_new	City elevation (hectometers)
AGE	Age (years)
