# Supplementary material for: Analyzing Personal Happiness from Global Survey and Weather Data: A Geospatial Approach
Source: PLoS One. 2016 Apr 14;11(4):e0153638. doi: 10.1371/journal.pone.0153638 (PMC4831775; doi:10.1371/journal.pone.0153638)
Supplement: S3 Table — (PDF) [file pone.0153638.s004.pdf]

S3 Table. Number of countries in this study and in the UN data

Regions	Num. of countries in survey data[1]	Total countries in the UN data[2]	Percentage
South America	1	14	7.1
Oceania	1	25	4
Northern America	1	5	20
Eastern Asia	2	7	28.6
South-Eastern Asia	1	11	9.1
Southern Europe	5	16	31.3
Western Asia	2	18	11.1
Eastern Europe	5	10	50
Northern Europe	5	18	27.8
Western Europe	5	9	55.6

Sources: 1.http://www.issp.org;

2.http://unstats.un.org/unsd/methods/m49/m49regin.htm.
